# Introducing the Fear Learning and Anxiety Response (FLARe) app and web portal for the remote delivery of fear conditioning experiments

**DOI:** 10.3758/s13428-022-01952-y

**Published:** 2022-09-07

**Authors:** T. McGregor, K. L. Purves, T. Barry, E. Constantinou, M. G. Craske, G. Breen, K. S. Young, T. C. Eley

**Affiliations:** 1 Social, Genetic and Developmental Psychiatry Centre, Institute of Psychiatry, Psychology & Neuroscience, King’s College London, London, UK; 2 NIHR Biomedical Research Centre for Mental Health, South London and Maudsley NHS Trust, London, UK; 3 Experimental Psychopathology Lab, Department of Psychology, The University of Hong Kong, Hong Kong, Hong Kong; 4 Department of Psychology, University of Cyprus, Nicosia, Cyprus; 5 Department of Psychology and Department of Psychiatry and Biobehavioral Sciences, University of California, Los Angeles, CA USA

**Keywords:** Fear conditioning, Classical conditioning, Pavlovian conditioning, Remote testing, Smartphones, Online research

## Abstract

Experimental paradigms measuring key psychological constructs can enhance our understanding of mechanisms underlying human psychological well-being and mental health. Delivering such paradigms remotely affords opportunities to reach larger, more representative samples than is typically possible with in-person research. The efficiency gained from remote delivery makes it easier to test replication of previously established effects in well-powered samples. There are several challenges to the successful development and delivery of remote experimental paradigms, including use of an appropriate delivery platform, identifying feasible outcome measures, and metrics of participant compliance. In this paper, we present FLARe (Fear Learning and Anxiety Response), open-source software in the form of a smartphone app and web portal for the creation and delivery of remote fear conditioning experiments. We describe the benefits and challenges associated with the creation of a remote delivery platform for fear conditioning, before presenting in detail the resultant software suite, and one instance of deploying this using the FLARe Research infrastructure. We provide examples of the application of FLARe to several research questions which illustrate the benefits of the remote approach to experiment delivery. The FLARe smartphone app and web portal are available for use by other researchers and have been designed to be user-friendly and intuitive. We hope that FLARe will be a useful tool for those interested in conducting well-powered fear conditioning studies to inform our understanding of the development and treatment of anxiety disorders.

## Introduction

### Background

Experimental paradigms are a fundamental methodology within psychological science. Laboratory-based experimental testing, however, is constrained by the significant time, financial costs, and manpower needed to conduct in-person assessments with individual participants. These constraints often limit how representative the study’s sample is and the overall sample size of the research. Together, these limitations harm the generalisability and replicability of experiments conducted in person (Ioannidis, 2005; Munafò et al., 2017). This is particularly relevant for fear conditioning research, a type of experiment that models critical processes involved in the development of, and recovery from, anxiety disorders. Fear conditioning experiments are typically conducted on samples of less than 100 participants (based on the most recent meta-analysis of patient-control differences; Duits et al., 2015). As such, the field of fear conditioning would benefit from larger and more heterogenous samples to ensure the replicability and generalisability of key findings.

Using remote experiments, researchers can collect much larger samples in shorter periods of time. Additionally, well-designed digital research tools can reduce the burden on researchers by making it easier to create, share, and configure psychological experiments. Such software can be released with source code (open-source software) to further increase access, enable replication, reduce duplication of effort, and encourage innovation and expansion.

Development of experimental research tools can take two different approaches, either non-specific or task-specific. Non-specific tools are highly adaptable, allowing researchers to create a range of tasks on a single platform (e.g. Gorilla; Anwyl-Irvine et al., 2020). Task-specific tools are created with a specific paradigm in mind and only allow flexibility that is relevant to the task whilst implementing control over important experimental parameters (e.g. SMARTA [Subject-Mediated Automatic Remote Testing Apparatus], RECAPP-XPR; Cortis Mack et al., 2019; Vagell et al., 2019). A key strength of non-specific tools is their flexibility, which allows adaptation of almost any experimental task into an easily deliverable online format. However, non-specific research tools often require the user to build their experiment using the command line, or without any paradigm-specific guidance. Alternatively, task-specific research tools can be developed in a user-friendly way that improves accessibility for researchers by providing a framework to build their experiment on. Whilst this approach inherently places more constraints on the user than non-specific research tools, it presents a compromise between flexibility and usability.

Here we describe the Fear Learning and Anxiety Response (FLARe) smartphone app and associated web portal, a set of tools designed for conducting fear conditioning research remotely. A key priority of the FLARe project was to provide an open-source, user-friendly platform to provide access to a validated remote fear conditioning paradigm. As such, we opted for a task-specific approach. Overall, creating a semi-flexible, remote research tool has the advantage that many of the technological and scientific issues involved in setting up, programming, and managing tasks are addressed by the developers. This approach streamlines the process of study design, implementation, and data acquisition for other researchers.

### Fear conditioning

Fear conditioning is a widely used experimental paradigm that models the development, maintenance, treatment, and relapse of anxiety disorders (Craske et al., 2018; Mineka & Oehlberg, 2008). Meta-analyses and large-scale experiments have found reliable associations with anxiety disorders that replicate across cohorts (Duits et al., 2015; Lissek et al., 2005; McGregor et al., 2021). Basic fear conditioning paradigms consist of two phases known as ‘acquisition’ and ‘extinction’. Acquisition models the development of fears via direct learning experiences, whereas extinction models the subsequent learning of safety and is considered to be a central mechanism of exposure-based treatments. Additional components of fear conditioning paradigms tap into other aspects of fear learning, like generalisation and return of fear (often assessed with renewal or reinstatement paradigms, Vervliet et al., 2013). A fear conditioning experiment typically consists of some combination of these experimental phases designed to assess different aspects of anxiety-related behaviour and responding.

**Fig. 1:**
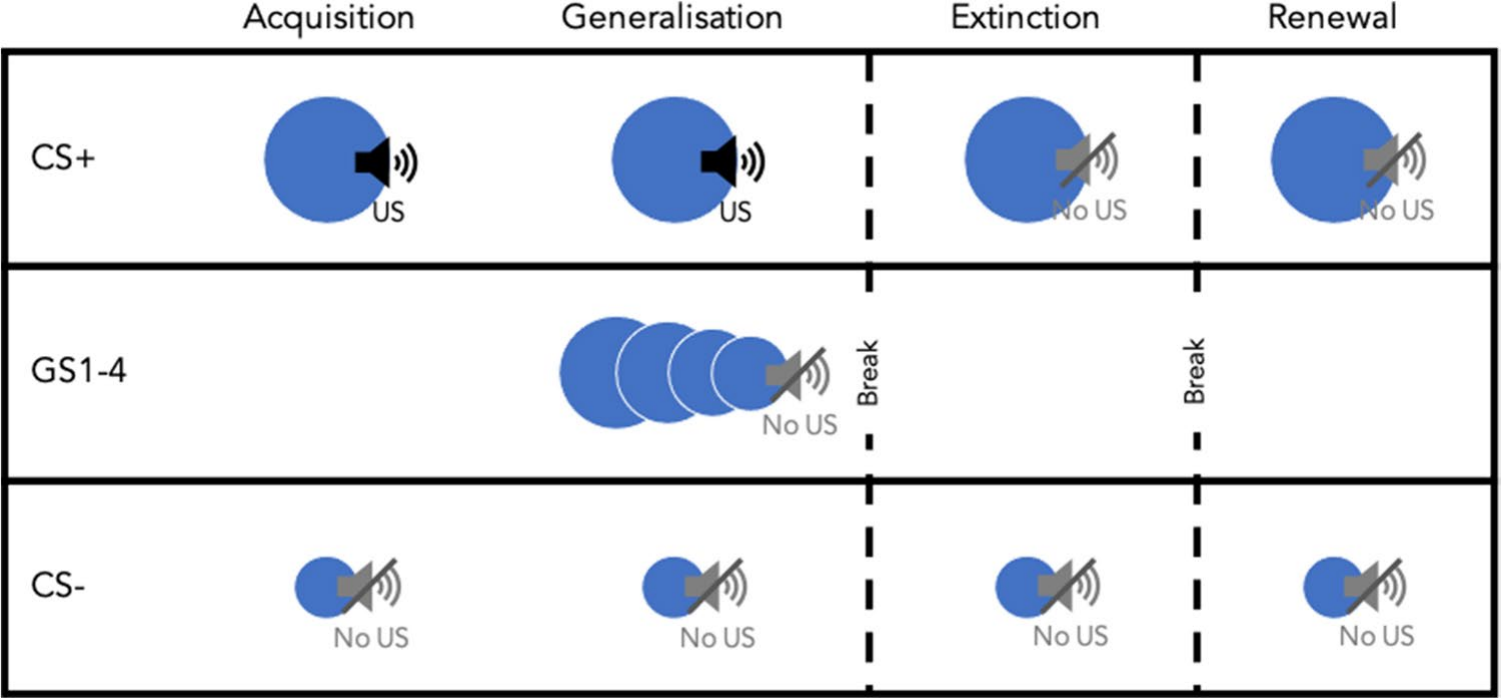
The stages of fear conditioning. CS = conditional stimulus, GS = generalisation stimulus, US = unconditional stimulus.

The FLARe app allows the design of fear conditioning paradigms relying on principles of Pavlovian conditioning to create associations between different stimuli (Fig. 1). Four phases are currently available to use in the FLARe app: acquisition, extinction, generalisation, and renewal. In *acquisition*, participants are presented with neutral ‘conditional stimuli’ (CS; e.g. images of different-sized circles). One CS is repeatedly paired with an aversive ‘unconditional stimulus’ (US; e.g. an unpleasant loud noise), while the other is always presented alone. Through repeated trials, participants learn to associate one of the CSs with threat due to its pairing with the US. This CS is known as the ‘CS+’. The other CS, not paired with the US, becomes associated with safety and is known as the ‘CS−’. During the *extinction* phase, both CSs are repeatedly presented in the absence of the US. Extinction leads to new learning, with the CS+ now holding competing meanings of threat and safety (Bouton, 1993). Throughout each stage, a participant’s ability to inhibit their fear response to a safety cue (CS−) relative to a threatening cue (CS+) gives an index of ‘discriminant conditioning’.

Two additional phases can be used in combination with acquisition and extinction to explore other anxiety-related processes: generalisation and renewal. The *generalisation* phase models how fear learning transfers to stimuli with similar perceptual or conceptual properties to the CS+ and CS− (Dymond et al., 2015). Generalisation of fear learning to different-sized circles could be assessed by presenting multiple circles that range in size between the circles used for the CS+ and CS−. Generalisation is assessed immediately after acquisition, prior to extinction. *Renewal* models the return of fear after treatment and is typically assessed at least 24 hours after acquisition and extinction take place (Lonsdorf et al., 2017). During the renewal phase participants are presented with the CS+ and CS− again, without the US (similarly to the extinction phase). This phase tests the extent to which participants retrieve their extinction learning over an extended period of time.

Fear conditioning can be quantified in a number of ways by measuring physiological, behavioural, self-report, and neurobiological responses. Specifically, skin conductance responses (physiological), avoidance (behavioural), expectancy ratings (self-report), and functional neuroimaging (neurobiological) are typical outcome measures in the field of fear conditioning (for more detail see Lonsdorf et al., 2017).

### FLARe rationale

The FLARe software was developed by a team based primarily at King’s College London to remotely deliver a fear conditioning task. Our primary research aim was to facilitate large-scale data collection necessary for evaluating genetic and environmental influences upon fear conditioning. Given the potential for ‘big data’ to improve the reliability of psychological research (Adjerid & Kelley, 2018), and the pivotal role of open and reproducible scientific practices, a second key aim was to create a platform available to other researchers without the necessity for advanced programming expertise or high budgets.

#### Choice of delivery platform

Remote tasks can be completed by participants on computers, tablets, or smartphones. Though many platforms exist to support the development of computer-based tasks (e.g. Anwyl-Irvine et al., 2020; de Leeuw, 2015; Gureckis et al., 2016; Sochat et al., 2016) the proportion of the population that owns a smartphone is increasing and has overtaken laptop/desktop ownership in some countries (e.g. the United States; Pew Research Center, 2021). Additionally, smartphones have a range of available functionality (e.g. passive data monitoring of app state, phone volume, and in-built notification flags) that can be used for a priori and post hoc control of the remote testing environment. For these reasons, we developed a smartphone app to enable remote experiment delivery to participants and a partner web platform (portal) for experiment setup and data processing for researchers.

#### Choice of outcome measures

We selected self-reported fear conditioning outcomes rather than physiological outcome measures as the former require no additional hardware or software and are a valid measure of fear conditioning (Boddez et al., 2013). The self-report measures most used in fear conditioning tasks are (1) expectancy ratings (how likely the CS is to be followed by the US during each trial), (2) affective ratings (the level of valence, arousal, or fear felt towards the CS after each phase), and (3) contingency awareness (ability to detect the association between the US and the CS+). These measurements assess different dimensions of fear learning (Lonsdorf et al., 2017). Expectancy ratings and contingency awareness measure explicit knowledge of the associations between stimuli, while affective ratings measure self-reported emotional responses to the stimuli (Baeyens & De Houwer, 1995).

## Overview of FLARe

**Fig. 2:**
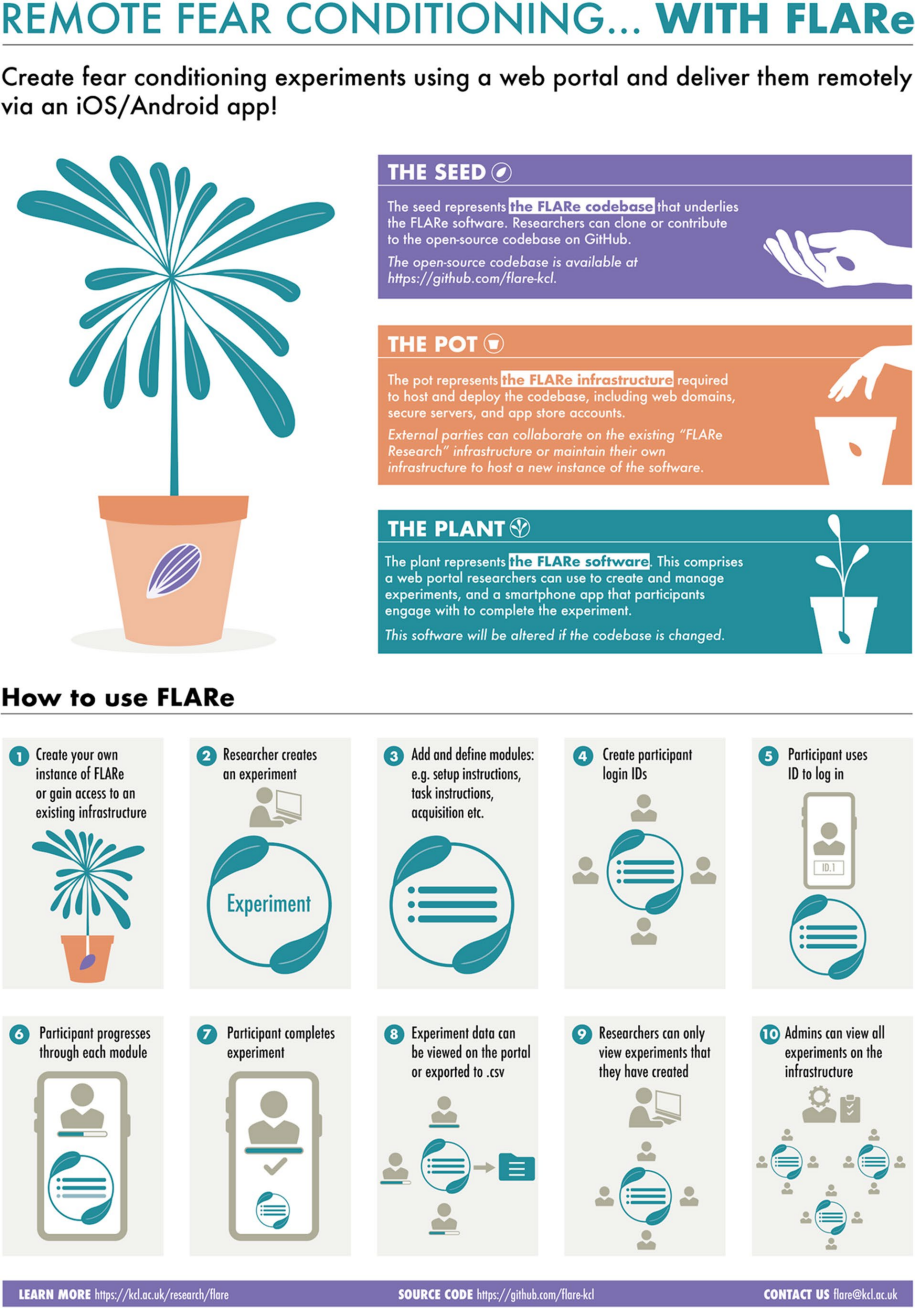
Infographic for understanding the different aspects of FLARe (codebase, infrastructure, software) and a researcher’s journey from creating an experiment to collecting and exporting data.

**Fig. 3:**
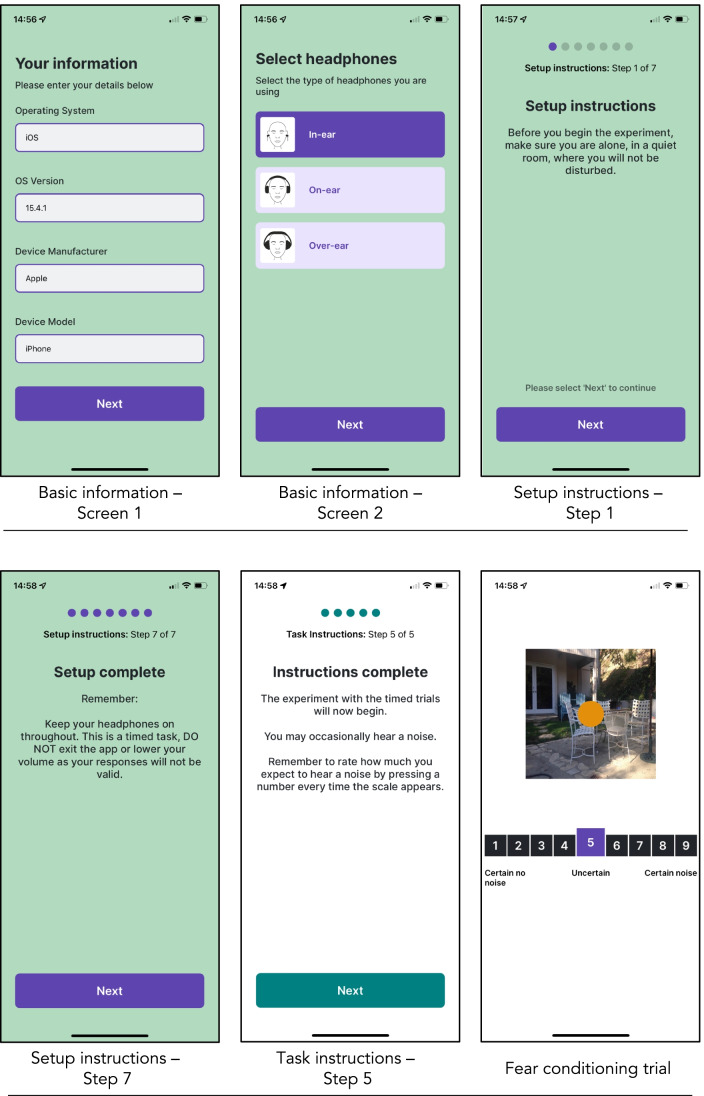
A selection of screenshots from different modules in the FLARe app. Modules unrelated to the fear conditioning task have a green background (basic information and setup instructions), whereas modules related to the task have a white background (task instructions and fear conditioning trials).

The FLARe software includes a smartphone application and web portal that can be used in tandem to deliver a range of flexible fear conditioning experiments (Fig. 2). This allows researchers to use one app to deliver numerous different versions of this paradigm. Participants download and use the app to access experiments using a unique login ID, which identifies the participant and the specific experiment in which they are taking part (see Fig. 3 for screenshots of the app interface). This login ID serves as the link between app and portal. Researchers use the portal to create and configure their experiments, generate participant login IDs, and view/export data from the app. The FLARe portal offers two levels of access to users: admin and researcher. *Admin users* have access to all experiments that exist on the portal whereas *researcher users* are only able to view and manage specific experiments and associated data that they created or have been added to.

**Fig. 4:**
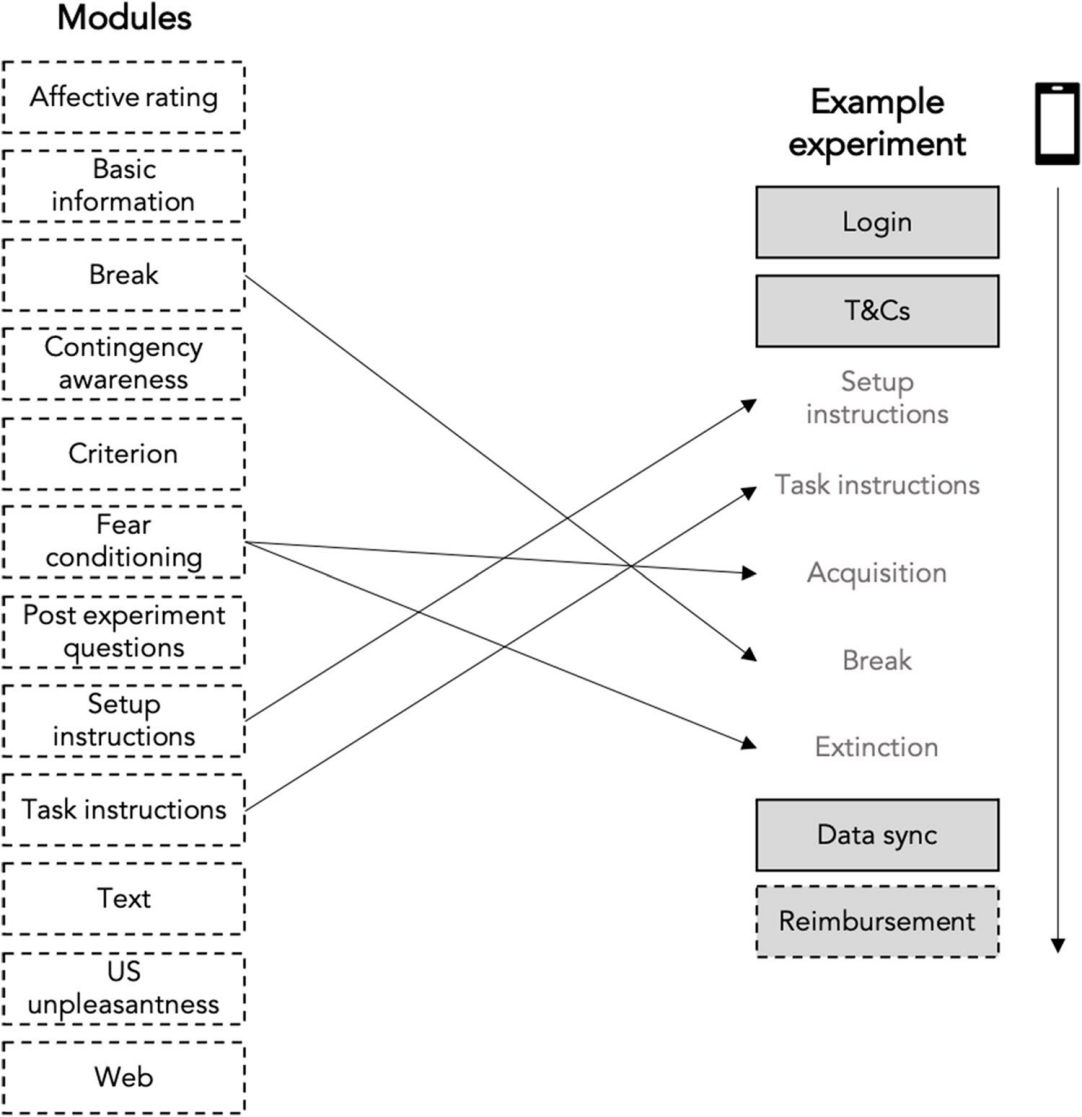
Illustration of how modules can be used to build FLARe experiments. All fully flexible modules are listed on the left, with an example of how they can be used to create a short experiment displayed on the right. Boxes with a solid border indicate mandatory modules that are always included in the study procedure. Grey boxes indicate modules with a fixed position within an experiment. Arrow to the right indicates a participant’s journey through the experiment once they have installed and opened the app.

Experiments are initially created by setting several experiment-wide parameters (Table 1). For example, researchers are required to upload their own CS image files and a US audio file to be used throughout the experiment. Once these are set, experiments are built using a variety of configurable modules (Table 2). Each module is designed with a specific purpose, such as delivering task instructions (task instruction module) or delivering fear conditioning trial screens (fear conditioning module). Each module has modifiable elements according to its purpose. For example, the fear conditioning module allows the number of trials and the rate of reinforcement (the number of times the CS+ is followed by the US) to be determined by the researcher. Modules broadly support three functions: (1) experiment delivery, (2) experiment management, (3) external integration. Modules can be placed anywhere within an experiment and used multiple times if needed. This framework means experiments can range from short one-day experiments that only collect acquisition and extinction data (see Fig. 4), to longer multi-day experiments that include multiple fear conditioning phases. There are also optional modules to facilitate data collection in-app, such as consenting and screening procedures and delivery of reimbursement via voucher codes after completion, as well as options to add links to additional measures delivered via external survey platforms. Currently, content on the FLARe app and portal (such as instructions) are only available in English.

**Table 1:** Global experiment settings

Setting	Description
Experiment code	Maximum six-character string associated with the experiment that is used as the prefix for auto-generated participant IDs.
Voucher pool	Researchers can choose to associate their experiment with a pool of voucher codes uploaded to the portal. If an experiment has a voucher pool, a reimbursement screen at the end of the experiment will provide participants with a voucher code.
Expectancy rating anchors	Character strings that are placed at the beginning, midpoint and end of the expectancy rating scales during each trial. These can be changed to reflect your chosen US.
Trial length	The total duration of a fear conditioning trial, defined in seconds.
Rating delay	The delay between the beginning of a fear conditioning trial and the appearance of the expectancy rating scale. This will determine the length of time that the CS and context appear without anything else on the screen.
ITI min/max delay	The lower and upper duration limits for the inter-trial intervals (ITI), in seconds. Any one inter-trial interval will last for a random number of seconds between these upper and lower limits (inclusive).
US	The unconditional stimulus, uploaded as a .wav file. If audio files last longer than one second, the app will trim the file so that only the first second of audio is used.
Minimum device volume	The minimum volume that a participant can lower their phone to without receiving a warning notification. This volume is determined as a proportion of the maximum volume on any given smartphone.
CSA/CSB	The conditional stimuli that will serve as the experiment CS+ (reinforced stimulus) and CS− (unreinforced stimulus). Any .png images that are 800-pixel x 800-pixel in size can be uploaded. The image (CSA or CSB) allocated as the CS+ is randomly determined by the portal for each new participant. Output is standardised to include both the stimulus name (CSA or CSB) and stimulus type (CS+ or CS−) for each participant.
GS A–D	Up to four stimuli that will serve as the experiment generalisation stimuli (uploaded as 800-pixel x 800-pixel .png files). The GSA should be most similar to the CSA, and the GSD should be most similar to the CSB. When CS+ is allocated for each user, the generalisation stimuli are standardised to match, such that the GS1 will be the most similar to the CS+, and so forth. Researchers can use fewer than four stimuli if required.
Context A–C	Images that will serve as the context image for the experimental trials. Up to three different images can be uploaded as 800-pixel x 800-pixel .png files. You can later choose to use any one of these context images to serve as the background for fear conditioning trials. Context images are allocated to all trials within a particular fear conditioning phase (e.g. acquisition, extinction, generalisation).

**Table 2:** Full list of modules available on the FLARe app

Module	Description	Primary use cases
Affective rating	Allows the researcher to ask a question about CS and GS images (e.g. “how does this image make you feel”). Responses are collected on a nine-point Likert scale with modifiable anchors at points one, five, and nine (e.g. “calm”, “neutral”, “anxious”).	Delivering and capturing affective ratings
Basic information	Captures demographic (date of birth and gender), device (phone make and operating system), and headphone (type: in-ear/on-ear/over-ear) information. Demographic questions can be turned off to maximise anonymity of participants.	Collecting important information about participants and the equipment they are using to take part in the experiment
Break	Produces two modules, a “break start” and “break end”, for which researchers specify a minimum amount of time that must elapse between them. The two break modules can be placed around other modules to prevent participants from continuing to another section of the experiment too quickly.	Forcing minimum time limits between fear conditioning modules (e.g. between acquisition and extinction phases)
Criterion	Allows researchers to ask any number of yes/no questions (e.g. “do you wish to take part in this study?”). Researchers have full control over question wording and can specify whether the response should be forced (i.e. participants cannot continue without answering). Researchers can also specify a required answer, enabling participants to be screened out if their response excludes them from the study.	Consenting and screening participants before taking part in a study
Contingency awareness	Asks participants “Did you happen to notice whether you heard the *audible keyword* after seeing a certain *visual keyword*?” Participants who respond “yes” to the question are taken to a second screen presenting them with an image of both CS to select from. Researchers can modify the audible (e.g. “loud scream”) and visual (e.g. “circle”) keywords so that they match the stimuli used for their specific study.	Determine whether or not participants are contingency-aware (i.e. did they recognise which stimulus was the CS+)
Fear conditioning	Delivers a phase of a fear conditioning task (e.g. acquisition or extinction). Researchers can adjust the number of presentations per CS/GS, the number of times the CS+ is reinforced, which context image is used, and whether GS are included in the phase.	Delivering different phases of a fear conditioning task, such as acquisition, generalisation, extinction, or renewal
Setup instructions/calibration	Provides instruction screens that minimally ensure that the smartphone has headphones connected and the device volume is above a limit specified by the researcher. Further instructions can be provided by adding additional screens that allow free text editing. Researchers can also choose to include additional steps to calibrate the volume of the US to a subjective point of aversiveness.	Ensuring the participant’s device and environment is set up correctly for taking part in the experiment
Task instructions	Provides instruction screens related to the fear conditioning task itself (e.g. how to make an expectancy rating). Most text can be edited, and further instructions can be provided by adding additional screens that allow free text editing.	Ensuring the participant knows how to complete the fear conditioning task
Text	Allows researchers to add additional screens of text that can be formatted using Markdown.	Delivering additional instructions, guidance, or information to participants
Web	Allows researchers to seamlessly link participants to an external website within the app (e.g. to watch something or undertake questionnaires during a break).	Delivering additional tasks or self-report measures (e.g. through an online survey platform)
Post-experiment questions	Asks participants a set of questions about their behaviour during the experiment (e.g. “Did you remove your headphones during the task?”). Researchers can choose which, if any, of these questions to ask participants.	Determining task adherence
US unpleasantness	Asks participants “How unpleasant did you find the *audible keyword*?” Researchers can modify the audible keyword (e.g. “loud scream”) to match the stimulus used for their specific study. Responses are collected on a ten-point Likert scale ranging from “Not unpleasant at all” to “Very unpleasant”.	Determining the aversiveness of the US
Reimbursement	Provides participants with an alphanumerical code (e.g. an Amazon gift code) to use as reimbursement. Researchers can upload a spreadsheet containing batches of codes to a ‘voucher pool’ on the portal. To activate the reimbursement module, researchers must associate the experiment with the voucher pool in the experiment settings.	Reimbursing participants

### Architecture

There are three components to FLARe: the codebase, the software, and the infrastructure (Fig. 2). The FLARe *codebase* contains all the source code required to build the FLARe *software* (the app and portal). The FLARe codebase is open-source, meaning anyone can create their own instance of the software provided they have the *infrastructure* to do so (access to servers, web domain, and app stores). The FLARe team based primarily at King’s College London have set up their own infrastructure to create an instance of FLARe known as ‘FLARe Research’. Any future mention of FLARe Research refers exclusively to the FLARe team’s version of the app and portal.

Technical development of the current FLARe software was carried out by a digital project agency, Torchbox (torchbox.com) to the specifications of, and with active involvement from, the FLARe team. The FLARe app codebase was written in React Native, a JavaScript-based open-source mobile application framework that can be used for developing across both Apple and Android platforms to enable maximal participant reach. Based on the version of the app at the time of writing, 15 MB and 41 MB of device memory is required to download the app on iOS and Android operating systems, respectively. The FLARe portal was coded using Django, a Python-based open-source web framework. This approach facilitates scalable, well-documented, easy-to-read/access code that will enable future rounds of development and the possibility of open-source contributions from external parties. Finally, the portal-hosted database is managed using PostgreSQL to facilitate data addition, deletion, and searching capabilities, with a readable interface facilitated by the open-source dashboard user interface kit ‘Tabler’. For detailed technical descriptions of the FLARe suite, see the GitHub-hosted code repository (github.com/flare-kcl) and technical description of the project therein (github.com/flare-kcl/flare-app/blob/develop/paper.md).

FLARe software is open-source, meaning that anybody can set up their own infrastructure for conducting remote fear conditioning research if they wish to. However, in this case, researchers will be required to implement, maintain, and fund this infrastructure by themselves. For further technical details please refer to our GitHub code repository github.com/flare-kcl. Alternatively, researchers may choose to collaborate with the FLARe team at King’s College London to gain access to the ‘FLARe Research’ infrastructure. In cases where small pilot studies are being conducted, the FLARe team may be able to provide access to the FLARe Research infrastructure for free. Where external research groups wish to use FLARe Research for larger studies, or require additional features for the app or portal, the FLARe team may require funding to be allocated to support maintenance and development of the platform. The FLARe team is particularly interested in supporting studies that comply with open science practices. As admins of FLARe Research, the FLARe team can manage and monitor all experiments being conducted on the platform with collaborating researchers’ access limited to their own experiments.

### Data storage

The FLARe Research application is deployed on a secure Heroku cloud application, hosted, and managed within Amazon’s secure data centres (for more information see: heroku.com/policy/security). The FLARe Research portal website (flareresearch.com) uses a secure, encrypted transfer protocol for communicating across networks and is password protected. Experiment data can only be accessed by members of the research team conducting the study and a limited set of FLARe team administrators.

All researchers external to King’s College London who wish to use the FLARe Research infrastructure must sign data sharing agreements due to the data being stored on servers owned by King’s College London. In most cases, the external researchers will own the data collected for their study and King’s College London will act as a data processor of all data collected during the use of the app. Occasionally, if a team at King’s College London is carrying out the study, or if King’s College London is collaborating with an external institution, King’s College London will be named as a data controller. Data collected on the FLARe Research portal is not personally identifiable as responses are pseudonymised and mostly consist of expectancy/affective ratings for the fear conditioning task. Researchers have the option to collect their participants’ date of birth, gender, and device information, the combination of which is unlikely to identify their participants. All information regarding the handling of participant data is covered in detail, and provided to participants, in the FLARe Research Privacy Policy (flareresearch.com/privacy-policy).

Data from experiments on the portal can be exported as a compressed folder (.zip) that contains one file (.csv) per module type. For example, data for all fear conditioning phases (acquisition, extinction, etc.) would be present in the fear conditioning module file, and data for all affective rating questions would be present in the affective rating module file. Participant, experiment, and module identifiers are present in each file to help merge data from different modules together.

### Remote experimental control

The quality of data collected using a remote fear conditioning task can be adversely affected by participants engaging in a number of unwanted behaviours: (1) removing/unplugging headphones; (2) lowering/varying the intensity of the US by adjusting the phone volume; (3) not engaging with the task (e.g. leaving the app); or (4) completing the task in a non-optimal location or situation. We have included various strategies to discourage these behaviours and track whether they occur.

#### Before the task begins (prevention)

A setup instructions module has been created that ensures headphones are attached and phone volume is appropriate for the study requirements. If these conditions are not met, the app prevents participants from continuing to the task. The setup instructions module also allows researchers to specify the optimal conditions for completing the task by adding additional instruction screens that allow free text editing. These might, for example, suggest sitting in a quiet room, where the participant will not be disturbed, ensuring their phone is fully charged and access to Wi-Fi is available.

#### During the task (monitoring)

Popup notification flags alert participants if they disconnect their headphones, lower their volume below a researcher’s predefined level, or if they fail to make a rating for more than two consecutive trials. During this time, the experiment is paused and cannot continue until the participant acknowledges the notification.

#### After the task is complete (post hoc control)

Volume change, failure to make a rating, and minimisation or exiting of the app are logged in participant data for the trial in which they occur. This enables data to be filtered post hoc based on metrics of participation and engagement. Whilst it is not possible to alert for or log instances in which participants physically remove their headphones from their ears, this can be assessed via an optional post-experiment questionnaire module. During this, participants can be asked any or all of the following: where the task was completed, if any interruptions were experienced, whether headphones were removed from their ears at any point in the task, and if so, roughly when this occurred.

### Applications

FLARe has several demonstrable and potential applications, some of which are described below with reference to case studies where applicable.

#### Assessing validity of remote fear conditioning

The original application of the first version of the FLARe app was to demonstrate the validity of the remote delivery of the task compared to traditional laboratory-delivered experiments. We found no differences in trial-by-trial responding between the two modes of delivery, and high within-person correlations between key fear conditioning outcomes delivered remotely and in person (Purves et al., 2019). Subsequently our group used the FLARe app to collect data in 1146 participants, 41% of whom reported symptoms consistent with a likely anxiety disorder (McGregor et al., 2021). We found remarkably similar patterns of associations between fear conditioning outcomes and anxiety as meta-analyses of in-person experiments (McGregor et al., 2021). From these studies we have concluded that the remote delivery of fear conditioning via the FLARe app is sufficient to evoke fear responses that are comparable to in-person delivery of the experiment.

#### Making data collection easier

A fundamental application of the FLARe software is to facilitate data collection in large samples, unrestricted by who can physically attend a testing session. As mentioned in the introduction, small sample sizes can lead to underpowered studies with samples that are not representative of the underlying population. The effect of this is amplified in specialist groups (e.g. individuals with particular diagnoses or exposures), geographically dispersed groups (e.g. nationwide samples), or groups whose access may be restricted (e.g. hospitalised inpatients). With an effective remote experiment, researchers can more easily reach larger, representative samples, and specific groups, than would be possible running an in-person study from a laboratory. FLARe enables this for fear conditioning research, as has been demonstrated via a large-scale (*n* > 1000) study into anxiety-related differences (McGregor et al., 2021), and preliminary work in specialist groups, including inpatients with an eating disorder diagnosis (Lambert et al., 2021).

Through the collection of large samples, FLARe can also facilitate a change of focus within the field of fear conditioning towards the study of individual differences. Several criticisms have been levelled at the tendency to study average responding in groups on fear conditioning tasks (Lonsdorf et al., 2017). This focus on mean differences could mask underlying patterns of subgroup responding, or poorly describe individual response trajectories (Lonsdorf et al., 2017). With an increase in sample sizes, studies will have the power to measure individual differences in responding and gain a more nuanced understanding of different factors associated with fear conditioning. The increase in power will also allow researchers to employ more sophisticated methods of analysis and make use of more complex study designs such as longitudinal, genomic, twin, prediction modelling, and treatment response research. A study using FLARe has already been conducted in a sample of more than 2000 twins to examine the heritability of and genetic correlations between fear conditioning outcomes (Purves et al., 2021). Data collection for this study took approximately three months, demonstrating the ease with which large-scale research can be conducted when an existing cohort is employed.

#### Easy validation of specific study design parameters

Remote studies can be conducted rapidly due to participants being tested simultaneously. The burden on researchers conducting experiments in this way is, therefore, reduced. We hope that if fear conditioning experiments are easier, cheaper, and quicker to run, researchers might be encouraged to answer research questions that were previously limited by available resources. This could be particularly useful when attempting to rapidly validate methods or experimental parameters for future work. For example, researchers could easily assess the impact of changes to trial and ITI duration, or the effect of replacing visual (CS) or auditory (US) stimuli. Our team has already taken advantage of the ease with which remote experiments can be conducted to validate a previous version of the FLARe app against an in-person version of the task (Purves et al., 2019). This experiment employed a loud human scream as the US and different size circles as the CS (validated files can be made available to other researchers upon request).

## Discussion

### Summary

As stated in the rationale, we aimed to create a platform for assessing fear conditioning remotely that would improve data sharing and methodological standardisation. Both these aims have been met in the latest round of development, with scope to refine and develop the research tool further.

The FLARe app and web portal provide a remote alternative to in-laboratory fear conditioning, enabling a genuine advancement in the scale of fear conditioning data collection. To date, the FLARe app has been used to collect data from a total of over 2750 individuals. Data from approximately 2500 of these participants was collected in just three months, supported for most of that time by just one team member. In comparison, our prior pilot and validation work, using in-person testing, collected data from 231 participants in 15 months, using at least three full-time team members. For context, our total sample collected via the FLARe app is greater than the largest meta-analysis of fear conditioning (Duits et al., 2015), which includes 2185 participants. The largest individual study within this analysis included 270 participants (Norrholm et al., 2011).

FLARe also represents an opportunity to improve the accessibility—to both participants and researchers—and standardisation of fear conditioning across studies. Accessibility has been achieved by creating a graphical user interface (GUI) web portal that makes it easy for researchers to set up and track their fear conditioning experiment without any special coding or software skills. The web portal facilitates standardisation by only allowing the customisation of parameters that commonly vary across fear conditioning studies (e.g. reinforcement rate, trial duration, CS image or US sound), whilst holding other aspects of the task constant (e.g. visual design and user experience). Additionally, all parameter choices are tracked via the portal meaning that, in the future, if multiple research teams use the same infrastructure, it should be simple to identify studies using similar designs. Similarity across studies will be increased due to the inherent consistencies within the app, mostly owing to standardised user experience. This will broaden the potential for multi-group collaboration, as well as meta- and mega-analyses. The numerous applications of the FLARe app, such as collecting large, representative samples, facilitating novel study designs, and making it easier to validate extensions of the paradigm, should all help to expand on and improve the reproducibility of the field.

### Limitations

The fundamental disadvantage of conducting remote research is the inability to control all aspects of the testing environment and participant behaviour that would normally be limited or observed by the experimenter in the laboratory. For example, location, time of day, and external distractions may all interfere with the participant’s behaviour during the experiment. Several strategies can be employed to reduce the likelihood and detect the occurrence of such behaviours using FLARe (see “Remote experimental control” section above). Additionally, studies have shown that results between in-laboratory and remote versions of experiments are comparable (Casler et al., 2013; Purves et al., 2019), which suggests that these limitations are unlikely to have a significant impact on research outcomes when data is obtained remotely.

Another limitation of remote data collection is the greater risk of drop-out after starting the task. Because it is easier to reach more people, more rapidly, it is likely that the number of participants who complete the app will still be greater than the number that could have been obtained with complete data in a comparable laboratory set-up. More concerning, however, is that certain groups of participants may drop out of the experiment due to important characteristics, such as greater levels of anxiety (McGregor et al., 2021). These participants may be more likely to continue the task with a researcher present. To reduce the likelihood of dropout due to the aversiveness of the fear conditioning task, the FLARe team have developed several features for researchers using the FLARe app. These features include the ability to use an alternative aversive stimulus as the US and a module for participants to calibrate the volume at which the US is played. There is some evidence to suggest that the default US used to validate the FLARe app (a loud female scream; Purves et al., 2019) can result in a large proportion of dropout compared to other stimuli that are rated as equally aversive (Michalska et al., 2016; Shechner et al., 2015). Use of an alternative US may reduce the number who attempt to avoid the sound by removing their headphones or lowering the volume of their phone. Furthermore, sensitivity to the US varies amongst participants. Whilst the validated version of the FLARe app (Purves et al., 2019) forced all participants to set their phone volumes to 100%, the latest version includes an optional experimental module that allows each participant to calibrate the US based on their own subjective ratings of aversiveness. In this module, participants hear the US at different volumes, selecting a volume that they personally find to be aversive, but not intolerable. Using a calibration module to standardise the volume based on subjective ratings of aversiveness rather than absolute volume may also improve the tolerance of the US and reduce dropout. Whilst this approach reflects the calibration usually used for the electric shock that is often the US for in-person fear conditioning, it is important to note that this new feature is yet to be validated against in-person testing.

### Future development

There are several development plans for progressing and improving the FLARe software. These proposals will be developed to work alongside the FLARe Research infrastructure but will depend on funding and the priority of features for planned studies.

#### Templates and reproducibility

At present, researchers from different groups need to communicate closely to ensure that their paradigms are comparable. In future rounds of development, we hope to improve standardisation across studies by introducing user generated templates for sharing experiment design, setup, and materials (e.g. image and sound files) with other groups in a way that is immediately replicable.

#### Centralised and open database

FLARe’s ability to collect ‘big data’ on fear conditioning could be further improved by refining the web portal’s database. As it stands, certain portal users (admins) are able to view data from all studies conducted using a specific instance of the FLARe software, an arrangement that needs to be considered in ethical approval and data sharing agreements with collaborating groups. For FLARe Research, this is restricted to the FLARe team admins who require access to all studies to facilitate successful use of the infrastructure by collaborating groups. It would be theoretically possible, given appropriate informed consent and ethical agreement, for admins to combine and analyse comparable anonymised data collected across different studies and geographic regions. Researchers collaborating on the same infrastructure (e.g. FLARe Research) would, therefore, be able to combine datasets to improve sample size further. We hope to develop this capability further to create a formalised central database where fully anonymised versions of data from studies collected on the same infrastructure could be combined, viewed, and exported together. This data would then be accessible to any researchers upon submission of an appropriate secondary data request that demonstrated a clear hypothesis, research plan, and ethical approval. Preparatory work for this future development includes careful consideration of variables collected using the FLARe app, ensuring that little to no identifiable data is stored on the database, and that all studies conducted using the FLARe Research infrastructure meet appropriate ethical standards and are compliant with data processing requirements. Future research conducted using the FLARe Research infrastructure will be required to comply with open science practices so that our centralised database of fully anonymised data can be shared openly, with due consideration of data protection and the rights of participants.

#### Incorporating measurements across multiple modalities

As mentioned previously, emotional responses to fear conditioning can be measured on a number of levels (e.g. self-report, behavioural, physiological, or neurobiological; see Lonsdorf et al., 2017 for a review). FLARe’s reliance on self-report measures of fear conditioning is, therefore, one limitation we hope to address in the future. Aside from only measuring one level of ‘fear’, self-report outcome measures have been criticised for their susceptibility to subjective biases and their lack of direct comparison with animal research (Lonsdorf et al., 2017). Incorporating additional measures that tap into levels of fear that are not susceptible to subjective biases (e.g. physiological measures such as skin conductance response, fear-potentiated startle, or heart rate) is a priority for future rounds of development. The choice to adopt smartphone technology gives FLARe the advantage of being able to incorporate physiological measures by making use of the phone’s built-in sensors, while continuing to conduct research remotely. At this point, a heart rate measure is the most likely candidate for integration into the app, since it can be collected in a number of different ways (e.g. through the smartphone’s camera/light or via wearables; Bai et al., 2018; Coppetti et al., 2017; Hwang et al., 2019). We will also investigate the feasibility of incorporating behavioural measures of anxiety, such as measures of avoidance, in future developments of the app. We hope to continue expanding the number of measures available to the researcher, allowing the measurement of different levels and components of fear.

## Collaboration statement

Recent improvements to FLARe and its underlying codebase now mean that it is ready to be used by other researchers through open-source sharing of the software or collaboration with the FLARe team using the FLARe Research infrastructure. The FLARe team aims to improve reproducibility within the field of fear conditioning research by forming collaborations that will lead to large-scale multisite studies of fear conditioning in line with a manifesto for reproducible science (Munafò et al., 2017). The FLARe team is open to discussing models for collaboration with interested groups and encourages all enquiries or questions.

## Concluding remarks

The FLARe smartphone app and web portal provides researchers the unique opportunity to conduct fear conditioning research remotely. These tools have been created with other researchers in mind, prioritising flexibility in study design while limiting other aspects of customisation for ease of use. Without the usual barriers associated with laboratory testing, researchers can use FLARe to carry out large scale, comparable fear conditioning studies with easily reproducible methodologies. FLARe holds the potential to collect ‘big data’ that could open the field of fear conditioning research to unprecedented sample sizes, offering enhanced reproducibility, reliability, and the power to examine nuanced individual differences in individuals with anxiety disorders.

## Data Availability

github.com/flare-kcl

## Declarations

### Open practices statement

An example dataset and validated stimuli files can be made available upon request by emailing flare@kcl.ac.uk. All code is available at github.com/flare-kcl. This manuscript summarises a novel instrument for research as opposed to findings from new research and, as such, was not preregistered.
